# Impact of flipped classroom strategy and learning on the acquisition of basic skills among students in the basketball 1 course at Birzeit University

**DOI:** 10.3389/fspor.2025.1660007

**Published:** 2025-11-24

**Authors:** Iyad Yousef, Mahmoud Kayed, Sana Liftawi, Shahd Hamdan, Jamal Alnuaimi, Hashem Al-Kilani

**Affiliations:** 1 Birzeit University, Birzeit, Palestine; 2 College of Education, United Arab Emirates University, Al Ain, United Arab Emirates; 3 School of Science, The University of Jordan, Amman, Jordan

**Keywords:** flipped classroom strategy, learning-through-play strategy, basic basketball skills, Birzeit University, students

## Abstract

**Introduction and research question:**

The aim of this study is to examine the impact of the flipped classroom strategy and learning through play on the acquisition of basic skills among students in the Basketball 1 course at Birzeit University.

**Theoretical framework:**

Theoretically, this study contributes to the literature on integrating technology in sports education, opening new avenues for understanding its impact on motor skill development. It also enriches the scientific understanding of modern educational strategies, such as flipped classrooms and gamified learning, and provides valuable data to guide the development of future educational programs.

**Methods:**

An experimental method was employed, with a sample of 66 male and female students divided into three equal groups of 22 students each. These included a control group taught using the traditional method, the first experimental group, which utilized the flipped classroom strategy, and the second experimental group, which applied the learning-through-play strategy. Data analysis was conducted using the Statistical Package for Social Sciences (SPSS).

**Results:**

The key findings revealed statistically significant differences favoring the flipped classroom strategy, followed by the learning-through-play strategy, for all basketball skills under investigation: chest pass, jump shot, lay-up shot, and dribbling. This study recommends incorporating both the flipped classroom and learning-through-play strategies in teaching fundamental basketball skills in the Basketball 1 course. To gather experimental data for analysis, the researchers observed the participants once a week.

**Implications:**

The findings suggest several significant implications: Projective learning is the second-best approach in the flipped classroom, second only to the traditional flipped classroom technique. Consequently, the flipped classroom approach is the recommended method for integrating physical education, particularly basketball, into Palestinian institutions and universities.

**Discussion and conclusion:**

This study contributes to filling research gaps in the literature by presenting a comprehensive approach that combines two modern strategies, namely, flipped learning and game learning, to improve fundamental motor skills in basketball, with a particular focus on the psychological and social effects, as well as the long-term impact of these strategies. This study expands the application of the flipped classroom approach in the field of motor skills in basketball, enriching the relevant literature on the flipped classroom mode.

## 1 Introduction

Owing to the rapid advancement of technology, integrating technological tools into sports has become necessary for enhancing performance and developing physical and technical skills. This transformation encompasses all sports, including basketball, in which significant advancements have been made in training and educational methods. Coaches and educators have adopted innovative techniques and modern tools to improve training quality. This reflects the growing importance of utilizing modern technology to educate students and athletes and teach them to use these tools effectively in sports. This need became especially evident during the pandemic when traditional education faced unprecedented challenges, particularly in practical areas such as physical education. In response, multimedia-based teaching methods have emerged, including illustrations, interactive videos, and audio-visual graphics. These tools play a crucial role in enhancing practical skill learning.

In recent years, the flipped classroom (FC) model has gained popularity (44, 45). Nevertheless, few studies have demonstrated how applying this paradigm improves academic performance, particularly in the field of physical education and, more specifically, primary education. Marqués et al. (1) compared the outcomes of a didactic proposal for teaching Korfball using an FC model with those of another group (the control), with an emphasis on the third cycle of Primary Education (fifth and sixth grades). Data was gathered using three different devices at three different times during the academic term. The findings indicate that students with FC performed much better in Korfball, particularly in terms of following the rules and using game strategies while playing. Regarding gender, Korfball appears to downplay the disparities in evaluation outcomes between boys and girls. Teachers who want to incorporate new ideas in the physical education classroom and researchers who wish to learn more about the advantages of FC and the use of Korfball in primary education may find this study helpful.

According to Chiang et al. (2), physical education is typically delivered in person because it places a strong emphasis on mastering sports skills. Teachers concentrate on outlining guidelines and modeling the motions that students must mimic and repeat. Since flipped classrooms have gained popularity, the current study examined their impact on physical education. A course that taught 3-on-3 basketball and used a variety of teaching techniques to test the effects of gender disparities on learning outcomes created and tested a mobile application for both beginner and expert basketball players. Students enrolled in the course above at a central Taiwanese institution were the participants. The study included 326 participants, comprising 238 men and 88 women. During the five weeks of the experiment, which took place in May and June 2017, the participants' ability to move correctly, maneuver, collaborate with others, maintain balance, and adapt was assessed and rated as learning outcomes. To collect empirical data for analysis, the study team observed the participants once a week. Projecting instruction is the second-best teaching method, according to the data; therefore, a mobile flipped classroom is the suggested method to include in physical education.

The basketball course benefited significantly from these efforts, as they helped improve student skill proficiency, thus elevating the overall quality of sports training (3, 4). In the Palestinian context, educational institutions face fundamental challenges that hinder the educational process, most notably the short duration of classes, unstable security conditions, and a lack of interactive teaching methods aimed at enhancing student motivation and engagement.

In traditional sports skills instruction, teachers often face challenges in motivating students and encouraging effective skill acquisition (43). Through their work as lecturers in physical education courses at Birzeit University, the researchers observed a decline in skill performance levels among students in Basketball Course 1 (46, 48). They suspect that this may be due to the students' reliance on traditional teaching methods, where the teacher is primarily responsible for explaining and demonstrating the skills, and there are limited opportunities for practice due to constrained class time. Each lecture session is only 50 min long, held twice a week, and the course involves numerous basic skills that require significant time to master. Furthermore, the instability of security conditions has caused frequent disruptions in face-to-face learning. This situation creates a need for more active and effective teaching strategies, such as the flipped classroom and learning-by-play, to compensate for the lack of continuity in in-person instruction. The flipped classroom approach involves students learning new concepts at home through brief videos, whereas the learning-by-play strategy focuses on engaging students in active play, which fosters motivation. To address these challenges, researchers decided to conduct a study comparing these two strategies under the exceptional circumstances of the current educational environment.

The importance of this study is highlighted in the following ways: (1) It responds to the urgent need for developing more effective teaching methods in practical courses at Birzeit University. (2) It introduces two modern teaching strategies for practical basketball skills at Palestinian universities. (3) It opens new avenues for researchers and academics to explore the use of these strategies in teaching other practical skills. (4) The study provides valuable data and insights on the effectiveness of the flipped classroom strategy and gamified learning. (5) It contributes to understanding how mathematical skills can be taught effectively through distance learning in the current context of Palestinian education.

The study aims to achieve the following objectives: (1) To examine whether there are statistically significant differences (*α* ≤ 0.05) in the pre- and post-measurements of the effect of using the traditional program on learning basic basketball skills (chest pass, jump shot, straight shot, dribbling) among members of the control group. (2) To examine whether there are statistically significant differences (*α* ≤ 0.05) in the pre- and post-measurements of the effect of using the flipped classroom strategy on learning basic basketball skills (chest pass, jump shot, lay-up shot, dribbling) among members of the first experimental group. (3) To examine whether there are statistically significant differences (*α* ≤ 0.05) in the pre- and post-measurements of the effect of using the learning-by-play strategy on learning basic basketball skills (chest pass, jump shot, lay-up shot, dribbling) among members of the second experimental group. (4) To examine whether there are statistically significant differences (*α* ≤ 0.05) in the post-measurements of the effect of using the traditional program, flipped classroom strategy, and learning-by-play strategy on learning basic basketball skills (chest pass, jump shot, lay-up shot, dribbling) among members of the control group, the first experimental group, and the second experimental group.

Theoretically, this study contributes to the literature on integrating technology in sports education, opening new avenues for understanding its impact on motor skill development. It also enriches the scientific understanding of modern educational strategies, such as flipped classrooms and gamified learning, and provides valuable data to guide the development of future educational programs.

Practically, the study provides solutions to enhance the teaching of basketball skills, enabling educational institutions to overcome time and spatial constraints that hinder the learning process. It also presents a practical framework for educators to enhance the learning experience, particularly in the challenging circumstances faced by Palestinian students. Additionally, it contributes to the development of more capable generations who can effectively utilize modern technologies to achieve educational and athletic goals.

Despite advancements in sports education, many interactive strategies for teaching motor skills still fail to adequately address individual differences or the specific needs of target groups. This gap has resulted in limited effectiveness for some strategies.

Strelan et al. (5) noted that effective education depends on the interaction between the student and their environment, which supports their holistic development and aligns with their interests and desires while facilitating knowledge acquisition. In this context, the flipped classroom strategy has proven effective. According to Verleger and Bishop (6), this strategy promotes student-centered learning by providing pre-recorded digital content, which allows for more time for in-class discussions and practical application. Its success relies on addressing individual student needs, making it a valuable alternative to traditional lectures. The learning-through-play strategy also proves beneficial (7). emphasized that play is a natural activity that enables students to explore their abilities and acquire skills in a fun and interactive manner. This strategy fosters the development of both mental and physical skills while creating a motivating atmosphere, making it particularly effective for teaching sports skills, such as basketball.

The objective of this research was to investigate how students enrolled in Birzeit University's Basketball 1 course acquired fundamental skills related to the flipped classroom approach and play-based learning.

This study aims to make a unique contribution by bridging research gaps in the literature, presenting a comprehensive approach that combines two modern strategies. This contribution involves developing new techniques. Firstly, the first strategy focuses on flipped learning and learning through games to improve fundamental motor skills in basketball. Secondly, the second strategy focuses specifically on the psychological and social effects, as well as the long-term impact of these strategies. By providing a thorough method that integrates two contemporary techniques—flipped learning and game learning to enhance basic motor skills in basketball, this study contributes to bridging research gaps in the literature. It focuses on the psychological and social impacts of these techniques as well as their long-term effects. This study expands the application of the flipped classroom in the field of motor skills in basketball and enriches the relevant literature on the flipped classroom mode.

The rest of the paper is structured as follows. The prior literature review is covered in Section 2. The sample and study methodology are presented in Section 3. Section 4 presents the discussion and empirical results. Section 5 concludes with a summary of the findings, policy implications, suggestions, and future research prospects.

## 2 Literature review and hypotheses development

### 2.1 Literature review

Recent literature highlighted the effectiveness of flipped learning as a modern educational tool that enhances the learning of motor skills and increases student engagement. Dai and Gao (8), Hatami and Savash (9), and Chen et al. (10) have shown that flipped learning improves comprehension of technical concepts and motor performance compared to traditional learning. These studies also found that the use of digital videos and pre-recorded content boosts student engagement and focuses on practical application, making flipped learning an effective approach in sports education Cheng et al. (11); Hu et al. (12); and Wei et al. (13) emphasized the importance of integrating digital technology with flipped learning. They found that digital platforms like MOOCs and WeChat facilitate self-learning and group interaction, which lead to improved technical skills and adaptability to modern learning methods. These findings highlight the additional benefits of utilizing technology to enhance flipped learning, making it more interactive and adaptable.

Hew and Lo (14), Dikilitas and Fructuoso (15), and Zhou et al. (16) have provided innovative models for advancing sports education through digital tools. They focused on creating interactive e-learning modules and 3D movement recognition technologies, which improved students' understanding of both theoretical and applied basketball concepts. Wang et al. (17) and Mason et al. (47) highlighted the importance of blended learning, which combines traditional and digital education, to enhance students' motor skills and sporting attitudes, thereby supporting its potential as an integrative model in sports education.

In a physical education teacher education program, Koh et al. (18) investigated how preservice teachers felt about a basketball course that employed a flipped learning approach. Eight preservice physical education teachers (three females; Mage = 23.5 years) who had finished the course participated in semi-structured interviews. They used both deductive and inductive theme analysis to code the interview material. Findings: Six key themes emerged that reflected the advantages, difficulties, and suggestions of flipped learning: supporting student-centered learning, encouraging self-directed learning, facilitating real-world application, the lack of adequate means to evaluate comprehension, the excessive time required for preclass preparation, and the need for alterations to materials and structure. In sport-based physical education teacher education courses, flipped learning may boost preservice teachers' enthusiasm for learning and improve active learning time. For physical education teacher education educators to successfully plan and implement flipped learning-based courses, the issues and suggestions they have found are helpful.

Koh et al. (19) examined the perceptions of preservice physical education teachers regarding a flipped learning basketball course in a physical education teacher education program. Semi-structured interviews were conducted with eight preservice physical education teachers (three females; Mage = 23.5 years) who had completed the course. Interview data were coded using inductive and deductive thematic analysis. Six main themes were identified reflecting the benefits, challenges, and recommendations of flipped learning: (a) facilitate student-centered learning, (b) promote self-directed learning, (c) encourage real-world application, (d) insufficient avenues to assess understanding, (e) pre-class preparation too time-consuming, and (f) modification of materials and structure. Flipped learning can potentially enhance preservice physical education teachers' motivation for learning and increase active learning time in the sport-based courses in physical education teacher education. The identified challenges and recommendations are valuable for physical education teacher education educators to prepare and effectively execute flipped learning-based courses.

Zhang et al. (20) built a research model based on a literature review. This model is used to analyze the influence factors of flipped classrooms on the learning effect of college students' motor skills. Through questionnaire survey and empirical analysis, we verified the research hypothesis of each factor. The results show that video technical action display, video feedback of students' technical action, and teaching interaction have a significant positive impact on the learning effect of college students' motor skills in the flipped classroom. In contrast, video-based teaching has no considerable impact. Wei et al. (13) analyzed the implementation of the flipped classroom model in basketball physical education using the WeChat public platform. The research aimed to explore how this model impacts student learning by utilizing short videos and mini-lectures to engage students and enhance their interaction with the educational content. The findings showed that integrating WeChat into teaching improved student participation and increased learning efficiency.

Killian et al. (21) investigated the impact of the FLA and traditional instruction on instructor participation, instructional context, and moderate-to-vigorous physical activity (MVPA) among students in middle school physical education classes. Fifty-six male pupils from two seventh-grade classes at a low-SES suburban school participated. This research used the System for Observing Fitness Instruction Time (SOFIT). We used descriptive statistics to analyze the data. To forecast SOFIT results as a function of FLA vs. conventional training, linear regression models were employed. Class time in MVPA was much higher for students in the FLA class (*p* < 0.01).

According to regression models, FLA class participants would spend 55% more time in MVPA classes (*p* < 0.01). Models revealed that students in FLA classrooms would spend over 18 more minutes in MVPA than students in regular courses after controlling for variables (*p* < 0.01). When compared to standard education, the FLA may be a helpful lesson improvement technique for raising student MVPA and promoting PE opportunities for children in low SES schools. Although the findings of this study showed promise for employing FLA in PE, it is essential to consider its limitations. It is necessary to investigate the FLA more.

Ferriz-Valero et al. (22) evaluated how 284 Spanish secondary PE students understand the conceptual substance of the activity in this example, volleyball—after using an FC technique. Assessing the impact of this intervention on their motivation from a Self-Determination Theory (SDT) standpoint was the second goal. A quantitative methodology revealed favourable outcomes for pupils using an FC strategy in terms of the growth of independent motivation and cognitive learning. These outcomes demonstrated the applicability of the used methodology when contrasted with a control group that took a more conventional approach to physical education.

Hu et al. (12) explored the effect of “MOOC + flipped classroom” on the teaching design and application of basketball courses in colleges and universities from the perspective of education modernization to promote the development of students' core literacy and provide a more theoretical basis and practical support for the in-depth research and promotion of “MOOC + flipped classroom” teaching mode. This study adopts a quasi-experimental design to study the teaching of basketball courses in colleges and universities based on “MOOC + flipped classroom.” The experimental class employed a “MOOC + flipped classroom” teaching approach (34 students), while the control group used traditional classroom teaching (30 students). Before and after the 16-week intervention, the student's learning effects were measured by basketball skill level assessment, Cooperation Ability Scale for University Students, Utrecht Work Engagement Scale-student, and Self-regulated Learning Scale, and the data were analyzed by independent sample *t*-test and repeated-measures ANOVA. The results, compared with the traditional classroom teaching mode, show that the “MOOC + flipped Classroom” teaching mode is innovative in terms of teaching philosophy, resources, and methods, promoting the informatization of education and furthering the realization of education modernization. The “MOOC + flipped classroom”-based teaching design for basketball courses benefits students' basketball skill level, study engagement, cooperation ability, and self-regulated learning ability, thus effectively promoting the students' core literacy.

Dai and Gao (8) discussed the application and practice of flipped classrooms in college basketball public physical education courses, providing a theoretical basis and practical guidance for improving the teaching effect of basketball. In this context, as an essential platform for cultivating students' physical and mental health and all-around skills, college basketball public physical education course urgently needs to improve the quality by reforming teaching concepts and methods, especially in the context of the new era, the introduction of the flipped classroom model not only refreshes the traditional teaching methods but also effectively breaks the inherent limitations of the teaching mode.

Li et al. (23) investigated the application of a teaching model that combines cooperative learning and flipped classrooms in university basketball courses in China. By analyzing the advantages and disadvantages of the traditional basketball teaching model and students' satisfaction with the course, the necessity of implementing cooperative learning and flipped classrooms is proposed. The study planned in detail the implementation strategies before class, in the school, and after class and compared them with the control group through an experimental design. The experimental results showed that the new teaching mode demonstrated significant advantages in terms of learning outcomes, student satisfaction, and teacher evaluation.

Li (49), Hu et al. (12), and Wang et al. (17) have examined how digital algorithms and educational materials improve basketball learning by customizing education to meet students' needs. These tools have improved students' engagement with academic material and enhanced their understanding of technical concepts, emphasizing the role of digital innovation in improving the learning experience.

Hatami and Savaş (24) conducted their research during the 2023–2024 academic year with 30 students from the Physical Education Department at Gazi University in Turkey. Participants were divided into an experimental group that followed a flipped learning approach using instructional videos and information cards via WhatsApp and a control group that received traditional lessons. Statistical analyses showed a significant improvement in psychomotor skills among the experimental group, with the mean increasing from (2.81 ± 0.978) in the pre-test to (3.67 ± 1.080) in the post-test, with a statistically significant difference (t = 4.235, *p* < 0.05). The control group also showed improvement but to a lesser extent, increasing from (1.91 ± 0.774) to (2.46 ± 0.724) (t = 3.335, *p* < 0.05). The findings indicate that flipped learning was more effective in enhancing basketball technical skills compared to traditional methods, though both approaches remained beneficial.

Wibowo et al. (25) evaluated the impact of the flipped classroom approach on student engagement in basketball learning within physical education, health, and recreation programs at the university level. A total of 62 students were divided into two groups: an experimental group that received instruction using the flipped classroom method and a control group that followed traditional teaching methods. The results showed that students in the experimental group demonstrated higher levels of engagement in behavioral, emotional, and cognitive dimensions compared to the control group. This suggests that the flipped classroom has a significant impact on enhancing the learning experience and increasing student participation in basketball education.

Sun et al. (26) and Zhang et al. (27) investigated the impact of the flipped learning model, which integrates online education with offline collaborative learning, on enhancing students' ability to design basketball instruction. The research was conducted on students enrolled in a compulsory basketball course at a Chinese university, divided into an experimental group that used the blended flipped classroom approach and a control group that followed traditional instruction. The results showed that the experimental group significantly improved their instructional design skills compared to the control group, demonstrating the effectiveness of the flipped classroom model in enhancing lesson planning and teaching quality in physical education.

Sun (28) examined the integration of the flipped classroom model with “Internet +” technology in college basketball education, aiming to improve teaching quality and student engagement. The researchers examine how optimizing online learning algorithms can improve learning accuracy, testing this model across different academic levels. The findings indicate that incorporating digital technology within the flipped classroom framework enhances student participation, skill acquisition, and overall learning satisfaction.

Karabulut-Ilgu et al. (29) and Liu (30) explored the implementation of the flipped classroom model in public physical education teaching at Chinese universities, aiming to enhance teaching quality and student engagement. The research examines the theory, methods, and effects of this model, with a focus on how blended learning, combined with digital technology, can enhance the learning experience. Educational experiments suggest that the flipped classroom approach improves students' physical activity practice in class, as it allows for more time for hands-on exercises rather than traditional theoretical lectures. Additionally, using online learning resources enhances students' self-directed learning and motivation.

Jhang et al. (31) propose a peer assessment-based flipped learning (PA-FL) approach to improving students' tactical performance in physical education. A quasi-experiment was conducted in a university basketball course in Taiwan. Two classes of university students were recruited as participants in the flipped classroom using mobile technology. One class, with 33 students, adopted the MPA-FL approach, while the other, with 27 students, adopted the conventional flipped learning (C-FL) approach. The results showed that students using the MPA-FL approach significantly outperformed those using the C-FL approach in terms of basketball tactical performance. On the contrary, there were no significant differences between the two groups in terms of critical thinking tendencies or reflective thinking.

Caruso et al. (32) explore deep learning methodologies, including video-based, skeleton-based, and sensor-based recognition models, and their applications in coaching, officiating, and performance evaluation. The study “Advancements in Basketball Action Recognition: datasets, methods, explain ability, and Synthetic Data Applications” systematically reviews basketball action recognition (BAR) using artificial intelligence (AI) and computer vision. It highlights challenges such as dataset limitations, occlusions, and model generalization issues while emphasizing the role of synthetic data and explainable AI (XAI) techniques.

Yuldashev (33) investigates the integration of technology in physical education and sports training, focusing on how digital tools enhance teaching and learning. It reviewed various technologies, including fitness trackers, mobile applications, augmented reality (AR), virtual reality (VR), and online learning platforms. The findings indicated that these tools enhanced student engagement and performance by offering interactive learning experiences and providing instant feedback.

Yousef et al. (34) evaluate the impact of international players' involvement in the Palestinian Basketball League on team cohesiveness. The researchers developed a three-dimensional cohesiveness evaluation instrument using a descriptive-analytical approach, encompassing psychological, social, and team membership aspects. Thirty pieces total, evenly split among the dimensions according to player position, experience, and status, made up the tool. 84 Palestinian Basketball League players, ages 18 to 38, participated in the study. The results showed that team affiliation had higher ratings (3.50) than psychological cohesiveness (3.21), whereas cohesion was regarded as moderate (mean = 3.32). Players with less than five years and more than ten years of experience had higher levels of cohesiveness than those with six to ten years, according to experience-based differences. Guards and forwards benefited from positional disparities. Although there were no discernible differences in the social dimension, reserve players demonstrated greater cohesiveness in the psychological and team belonging aspects, particularly in terms of player status.

Flipped learning increases possibilities for teacher-student engagement in the classroom by moving the lectures that provide course material to pre-class time. As a result, there are more opportunities for students to engage with professors. However, researchers have noted that to encourage students to think critically and reflect, flipped classrooms must incorporate practical learning tools. The goal was to enhance students' tactical performance in physical education by employing a peer assessment-based flipped learning (PA-FL) approach.

Jhang et al. (31) carried out a quasi-experiment in a basketball course at a Taiwanese university. They used mobile technologies to recruit two groups of university students to participate in the flipped classroom. Thirty-three students in one class used the MPA-FL technique, and twenty-seven students in the other class used the traditional flipped learning (C-FL). The findings demonstrated that, in terms of basketball tactical performance, pupils who used the MPA-FL strategy fared noticeably better than those who used the C-FL technique. Conversely, there were no appreciable variations in the introspective or critical thinking styles of the two groups.

Despite these promising results, a gap remains in the literature regarding the long-term impact of flipped learning and digital technology, particularly in terms of its social and psychological aspects. Furthermore, many studies focus on specific groups or conditions, limiting the ability to generalize the findings.

Udvaros and Pšenáková (35) explore the role of automatic evaluation systems in enhancing the flipped classroom methodology, particularly in programming and technology education. The research highlights how these systems improve learning efficiency by providing instant feedback, thereby increasing student engagement and academic progress. The findings suggest that utilizing automatic evaluation tools enhances student motivation and learning outcomes, enabling them to learn at their own pace while using classroom time for interactive activities and complex problem-solving.

### 2.2 Hypotheses development

#### 2.2.1 First hypothesis

There are statistically significant differences (*α* ≤ 0.05) in the pre- and post-measurements of the effect of using the traditional program on learning basic basketball skills (chest pass, jump shot, straight shot, dribbling) among members of the control group. Previous literature, such as Hatami and Savas (24), indicates that traditional education yields improvements in motor skills, though to a limited extent compared to innovative teaching methods like flipped learning.

Wei et al. (13) found that traditional education often lacks interactivity, which can reduce students' motivation and comprehension of technical concepts. This hypothesis aims to verify the effectiveness of traditional education as a baseline for comparison with modern methods. Previous literature highlights the relevance of this hypothesis by providing an analytical measure of traditional outcomes, which serves as a reference for understanding the effectiveness of new strategies.

H1. There are no statistically significant differences (*α* ≤ 0.05) between the pre- and post-measurements of the control group taught using the traditional program in the acquisition of basic basketball skills (chest pass, jump shot, lay-up shot, and dribbling).

*(This hypothesis serves as a baseline for comparison and assesses the natural improvement resulting from traditional instruction.)*

#### 2.2.2 Second hypothesis

There are statistically significant differences (*α* ≤ 0.05) in the pre- and post-measurements of the effect of using the flipped classroom strategy on learning basic basketball skills (chest pass, jump shot, lay-up shot, dribbling) among members of the first experimental group.

Dai and Gao's (8) study confirmed that flipped education enhances the understanding of technical concepts and fosters greater student interaction compared to traditional education. Wibowo et al. (25) demonstrated that flipped learning improves emotional, cognitive, and behavioral engagement, which in turn boosts the effectiveness of motor skill learning. Hu et al. (12) demonstrated that combining flipped learning with digital tools such as MOOCs significantly enhances technical skills. This hypothesis seeks to test the effectiveness of flipped learning compared to traditional methods. The literature supports this by emphasizing that flipped learning offers a more interactive and immersive learning experience.

H2. There are statistically significant differences (*α* ≤ 0.05) between the pre- and post-measurements of the first experimental group taught using the flipped classroom strategy in the acquisition of basic basketball skills (chest pass, jump shot, lay-up shot, and dribbling), favoring the post-measurement.

*(This hypothesis tests the effectiveness of the flipped classroom model in enhancing students' motor performance and engagement compared to traditional methods.)*

#### 2.2.3 Third hypothesis

There are statistically significant differences (*α* ≤ 0.05) in the pre- and post-measurements of the effect of using the learning-by-play strategy on learning basic basketball skills (chest pass, jump shot, lay-up shot, dribbling) among members of the second experimental group. Parker et al. (36) confirmed that learning through play fosters an engaging learning environment, which contributes to the improvement of both motor and artistic skills.

Wei et al. (13) found that interactive activities, like gamified learning, enhance students’ comprehension and engagement with educational content. Mercanet et al. (37) focused on designing digital resources that combine with gamified learning to improve educational outcomes. This hypothesis examines the role of playful learning in enhancing motor skills and student engagement. The literature supports this by showing that gamified learning caters to students' motor and emotional needs, making it an effective approach for teaching sports skills.

H3. There are statistically significant differences (*α* ≤ 0.05) between the pre- and post-measurements of the second experimental group taught using the learning-through-play strategy in the acquisition of basic basketball skills (chest pass, jump shot, lay-up shot, and dribbling), favoring the post-measurement.

*(This hypothesis evaluates the impact of gamified learning in developing technical and psychomotor performance through play-based instruction.)*

#### 2.2.4 Fourth hypothesis

There are statistically significant differences (*α* ≤ 0.05) in the post-measurements on the effect of using the traditional program, the flipped classroom strategy, and the learning-by-play strategy on learning basic basketball skills (chest pass, jump shot, lay-up shot, dribbling) among members of the control group, the first experimental group, and the second experimental group. Hatami and Savas (24), Dai and Gao (8), and Wibowo et al. (25) indicated that flipped learning yields superior results compared to traditional education.

Dikilitas and Fructuoso (15) and Zosh et al. (7) confirmed that interactive strategies, such as gamified learning, enhance educational outcomes when compared to traditional methods. Wang et al. (17) found that blended learning, which combines multiple strategies, achieves the best results for motor skills and sports-related outcomes. This hypothesis aims to compare the effects of different teaching methods comprehensively. The literature supports this by highlighting the superiority of flipped and gamified learning over traditional education, with careful comparisons necessary to determine the most effective approach.

H4. There are statistically significant differences (*α* ≤ 0.05) in the post-measurements among the three groups (control, flipped classroom, and learning-through-play) in the acquisition of basic basketball skills (chest pass, jump shot, lay-up shot, and dribbling).

*(This hypothesis aims to determine which teaching strategy produces the most significant improvement in basketball skill acquisition among university students.)*

*“*These hypotheses were tested using experimental and inferential statistical methods (Paired t-test, ANOVA) to determine the effectiveness of each instructional approach.”

The hypotheses logically extend previous literature, providing an empirical framework to validate the findings of prior studies on the effectiveness of flipped learning and gamified learning compared to traditional education. They also address gaps in the literature, such as the impact of these strategies on both motor and social skills, making them complementary to the results of earlier research.

This study aims to address these challenges by exploring the effectiveness of two modern strategies: the flipped classroom, which redefines the learning process by shifting theoretical learning outside the classroom, thereby allowing more time for practical application, and the learning-through-play strategy, which creates an engaging, interactive learning environment that simulates the physical and mental needs of students.

Furthermore, the global context underscores the importance of adapting educational methods to align with technological development, which was particularly emphasized during the pandemic. This highlights the need for flexible and digital education. Therefore, this study seeks to fill a knowledge gap regarding how these strategies can improve the learning of basic basketball skills. In light of these objectives, the study holds both theoretical and practical significance.

Based on the objectives of the study and the review of previous literature, the following hypotheses were formulated to test the impact of different teaching strategies—traditional, flipped classroom, and learning-through-play—on the acquisition of basic basketball skills among students enrolled in the *Basketball 1* course at Birzeit University:

## 3 Methodology and procedures

The researchers employed an experimental method, which was best suited for the study's objectives.

### 3.1 Study population

The study population consisted of physical education students enrolled in the Basketball 1 course during the 2024–2025 academic year. A total of 81 male and female students were included, based on the course records from the Physical Education Department at Birzeit University.

### 3.2 Study sample

The study sample comprised 66 male and female physical education students enrolled in the Basketball 1 course during the second semester of the 2023–2024 academic year. These students were selected intentionally and divided into three equal groups: (1) A control group that received traditional instruction. (2) The first experimental group that used the flipped classroom strategy. (3) A second experimental group that applied the learning-through-play strategy. Each group consisted of 22 students. Table 1 provides a detailed comparison of the study sample's characteristics, including height, weight, age, and baseline basketball skills.

**Table 1:** Results of the one-way ANOVA for testing the equivalence among the three groups (*n* = 66).

Variables	Source of variance	Sum of squares of deviation	levels of freedom	Mean squares	Value (f)	level Connotation
The age	Between groups Within groups the total	2.21 43.50 45.71	2 63 65	1.107 0.69	1.604	0.209
Height	Between groups Within groups the total	117.94 2,005.59 2,123.53	2 63 65	58.97 31.835	1.852	0.165
The weight	Between groups Within groups the total	55.39 1,641.23 1,696.62	2 63 65	27.697 26.051	1.063	0.351
Chest pass	Between groups Within groups the total	2.38 36.13 38.51	2 63 65	1.190 0.574	2.074	0.134
Jump shot	Between groups Within groups the total	0.67 39.63 40.30	2 63 65	0.333 0.629	0.529	0.592
Lay-up-shot	Between groups Within groups the total	0.88 43.30 44.18	2 63 65	0.441 0.687	0.642	0.530
Interviewer	Between groups Within groups the total	0.21 22.29 22.50	2 63 65	0.103 0.354	0.29	0.749

Source(s): Authors’ work.

#### 3.2.1 Sampling design and participants

The study employed an intentional (purposive) sampling design, selecting participants who were officially enrolled in the *Basketball 1* course offered by the Department of Physical Education at Birzeit University during the second semester of the 2023–2024 academic year.

The total sample consisted of 66 male and female students, divided equally into three groups (22 students each): a control group, a flipped classroom experimental group, and a learning-through-play experimental group.

Participants' ages ranged between 19 and 21 years (M = 19.4, SD = 0.8), representing a relatively homogeneous group in terms of physical education background, academic level, and motor skill proficiency.

The purposive selection ensured that all participants had similar exposure to the basketball curriculum, which strengthened internal validity and minimized extraneous variation.

It is clear from Table 1 that there are no statistically significant differences at the level of significance (*α* ≤ 0.05) in the pre-measurement of the variables of age, height, weight, and basic skills in the game of basketball (chest pass, jump shot, lay-up-shot, dribbling) among the members of the study sample in the three groups. This means the equality of the study sample members before starting to apply the educational programs to each group.

#### 3.2.2 Statistical analysis (clarification of ANOVA Use)

To examine the differences among the three study groups (control, flipped classroom, and learning-through-play) in the post-test measurements of basketball skills (chest pass, jump shot, lay-up shot, and dribbling), a One-Way Analysis of Variance (ANOVA) was employed.

This test was selected because it allows for the comparison of mean differences among more than two independent groups under a single independent variable (teaching strategy).

Prior to running ANOVA, data normality and homogeneity of variance were verified using Levene's test to ensure the assumptions of parametric testing were met.

When statistically significant F-values (*p* ≤ 0.05) were obtained, Bonferroni *post-hoc* tests were conducted to identify which pairs of groups differed significantly.

Additionally, Eta-squared (*η*^2^) values were calculated to determine the effect size of each teaching strategy on the improvement of basketball skills.

This analytical approach was appropriate for testing Hypothesis 4, which aimed to compare the relative effectiveness of the three instructional strategies.

### 3.3 Sample's equivalence

Before starting to implement the educational program, equivalence was conducted between the members of the study sample in the three groups by pre-measurement of the variables under study through the use of the one-way analysis of variance test (One-Way ANOVA) to compare the pre-measurement averages of the variables of age, height, weight, and basic skills in the game of basketball (chest pass, jump shot, lay-up-shot, and dribble) among members of the study sample in the three groups. See Tables 2, 3 below.

**Table 2:** The means and standard deviations for the variables (age, height, and weight) of the study sample members (*n* = 66).

Variables	Unit of measurement	Control group (*n* = 22)		Flipped classroom strategy (*n* = 22)		Strategy by playing (*n* = 22)	
		Arithmetic mean	Standard deviation	Arithmetic mean	Standard deviation	Arithmetic mean	Standard deviation
age	year	19.60	0.85	19.18	0.86	19.53	0.78
height	right	175.68	5.02	178.95	5.71	177.23	6.14
weight	kg	67.50	4.69	69.05	5.14	69.68	5.45

Source(s): Authors’ work.

**Table 3:** The means and standard deviations for the pre-measurement of basic skills in basketball among members of the study sample in the three groups (*n* = 66).

Basic skills	Unit of measurement	Control group (*n* = 22)		Flipped classroom strategy (*n* = 22)		Learning strategy by playing (*n* = 22)	
		Arithmetic mean	Standard deviation	Arithmetic mean	Standard deviation	Arithmetic mean	Deviation Standard
Chest pass	second	12.39	0.78	12.86	0.70	12.62	0.79
Jump shot	level	16.54	0.77	16.30	0.75	16.36	0.86
Lay-up-shot	second	24.56	0.77	24.49	0.85	24.29	0.86
dribbling	second	12.88	0.48	12.95	0.68	13.01	0.61

Source(s): Authors’ work.

### 3.4 Validity and reliability of the tests used in the research

Although the tests used in this study were valid and standardized, the researchers calculated their validity coefficient by presenting them to a panel of experts with doctorate degrees in physical education, specializing in teaching and coaching basketball. These experts evaluated the suitability of the tests in relation to the study's goals and sample, and suggested any necessary adjustments. A list of the experts, including their qualifications, affiliations, and areas of expertise, was provided in Supplementary Appendix 5.

### 3.5 Reliability coefficient of the dependent variables

To determine the reliability of the motor skill tests used in this study (chest pass, jump shot, lay-up shot, and dribbling), the test–retest method was applied. The tests were administered twice to an exploratory sample of 15 students from the same population but not included in the main study, with a one-week interval between the two applications under identical conditions.

The Pearson correlation coefficient (r) was calculated for each dependent variable to assess measurement stability. The obtained values demonstrated high reliability for all tests, as shown below in Table 4.

**Table 4:** Measurement stability and readability.

Skill test	Reliability coefficient (r)	Significance level (p)	Interpretation
Chest Pass	0.87	*p* ≤ 0.01	High Reliability
Jump Shot	0.91	*p* ≤ 0.01	Very High Reliability
Lay-up Shot	0.89	*p* ≤ 0.01	High Reliability
Dribbling	0.93	*p* ≤ 0.01	Very High Reliability

Source(s): Authors’ work.

These results confirm that the measurement instruments used in the study possess strong internal consistency and temporal stability, ensuring the accuracy of the collected data.

### 3.6 Stability of study tools

The reliability coefficient of the tests (accuracy of the chest pass, jump shot test, lay-up shot test, and dribbling test) was calculated using a test-retest method. The tests were applied to an exploratory sample of 15 students from the same research population but not part of the study sample. The tests were first administered and then repeated after one week under the same conditions to assess the stability of the results.

#### 3.6.1 Tutorial for all groups

Supplementary Appendix 1 provides detailed information on the technical and practical aspects of each group's program. The researchers implemented the educational program for the Basketball 1 course according to the course plan over 8 weeks, with two educational sessions per week, each lasting 50 min. The program was structured as follows: (a) 5 min for warm-up (for all groups together) (b) 10 min for technical instruction (for each group separately) (c) 30 min for practical application (for each group separately). (d) 5 min for cool down (for all groups together).

#### 3.6.2 Steps to conduct the study

##### 3.6.2.1 Preparation of equipment and tools

All necessary equipment and tools for the study tests were prepared.

##### 3.6.2.2 Designing data collection forms

A form was designed to collect data and observations related to each student's performance on the basic skills tests.

##### 3.6.2.3 Designing educational lessons

Educational lessons for the flipped classroom and playful learning strategies were designed. Videos and lesson plans for the proposed skills were selected and reviewed by the panel of experts.

##### 3.6.2.4 Conducting pre-tests

Were administered to all three groups (control, first experimental, and second experimental) in the sports hall of Birzeit University. The tests used were appropriate to the research goals.

##### 3.6.2.5 Conducting a pilot study

A pilot study was conducted to assess the suitability of the procedures, tests, and educational units. A sample of 15 students was divided into three groups of five, each experiencing one of the educational methods: traditional, flipped classroom, and learning through play. After the pilot study, the sample was excluded from the main study.

##### 3.6.2.6 Application of the educational program

The educational program was applied to the control group under the same conditions as the experimental groups, including the number of sessions, duration (50 min per session), and the 8-week program (two meetings per week). The only difference was the teaching method used: traditional, flipped classroom, or Playful learning.

##### 3.6.2.7 Periodized training program structures

The study implemented an 8-week training program, consisting of two sessions per week (90 min each) for all groups. The structure and content were standardized in terms of time, load, and skill focus, but differed in teaching methodology according to group assignment (traditional, flipped classroom, or learning-through-play) are illustrated in Table 5 below.

**Table 5:** Training program structures periodization.

Week	Control group (traditional instruction)	Experimental group 1 (flipped classroom)	Experimental group 2 (learning-through-play)
1–2	Introduction to basic basketball rules and warm-up drills using instructor-led explanation and demonstration.	Pre-class videos and readings on basketball fundamentals; in-class discussion and guided practice.	Games introducing basketball rules and coordination-based play activities.
3–4	Practice of chest pass and dribbling through repetitive drills.	Flipped approach: pre-class analysis videos + peer feedback during practice.	Relay games and small-group competitions emphasizing passing and dribbling accuracy.
5–6	Teaching lay-up shot and jump shot through instructor-led demonstration and correction.	Pre-recorded tutorials and group self-assessment; instructor feedback during performance.	Modified games integrating lay-up and jump shot tasks under playful competitive settings.
7–8	Comprehensive review and formal assessment under teacher supervision.	Student-led sessions demonstrating acquired skills and reflective discussion on flipped learning outcomes.	Tournament-style games combining all skills in cooperative and competitive play contexts.

Source(s): Authors’ work.

The periodization aimed to ensure progressive skill acquisition and balanced physical load across all groups, with methodological differences emphasizing the instructional strategy rather than training volume.

##### 3.6.2.8 Implementation of the educational program

The educational program was delivered to all three groups between March 10 and May 10, 2024, spanning two months. Each group participated in 16 sessions (two per week), with each session lasting 50 min. The sessions were divided into three segments: introductory, main, and concluding parts.

##### 3.6.2.9 Conducting post-tests

Were conducted for the control and experimental groups between May 12 and May 15, 2024, in the sports hall of Birzeit University. The conditions for the post-tests were the same as those for the pre-tests, allowing for a comparison of the results.

### 3.7 Study variables

#### 3.7.1 Independent variables

Tutorial using traditional teaching methods. (b) Tutorial using the flipped classroom strategy (c) Educational program using the learning-by-playing strategy (See Supplementary Appendix 4).

#### 3.7.2 Dependent variables

Response to tests measuring basic basketball skills: (a) Accuracy of chest pass. (b) Accuracy of jump shot. (c) Accuracy of lay-up shot. (d) Dribbling accuracy.

### 3.8 Statistical analysis

To test the study hypotheses and achieve its objectives, the researchers will use the Statistical Package for the Social Sciences (SPSS) and apply the following statistical methods: a) Means and standard deviations. b) Pearson correlation coefficient. c) Paired t-test (for two related means). d) Independent t-test (for two independent means).

## 4 Results and discussion

### 4.1 First hypothesis results

The first hypothesis posits that there are statistically significant differences in the pre-and post-measurements of the effect of using the traditional program on learning basic skills in basketball (chest pass, jump shot, lay-up shot, and dribbling) among members of the control group. To test this hypothesis, a paired t-test was conducted to assess the significance of the differences in means between the pre-and post-measurements for the control group. The results are presented in Table 6.

**Table 6:** (*T*-test) results for correlated samples (Paired-t-test) To indicate differences in the effect of using the traditional program on learning basic skills in the game of basketball among members of the control group (*n* = 22).

Basic skills	Unit of measurement	Pre-measurement		Dimensional measurement		*t*-value	Significance level	Improvement rate%	Cohen's d
		Arithmetic mean	Standard deviation	Arithmetic mean	Standard deviation				
Chest pass	second	12.39	0.78	12.23	0.58	0.92	0.36	347.6	0.818
Jump shot	level	16.54	0.77	16.74	0.41	-1.19	0.24	96.9	0.797
lay-up-shot	second	24.56	0.77	24.11	0.94	1.56	0.13	171.4	1.349
dribbling	second	12.88	0.84	12.75	0.95	0.63	0.54	93.3	0.93

Source(s): Authors’ work.

**Figure 1:**
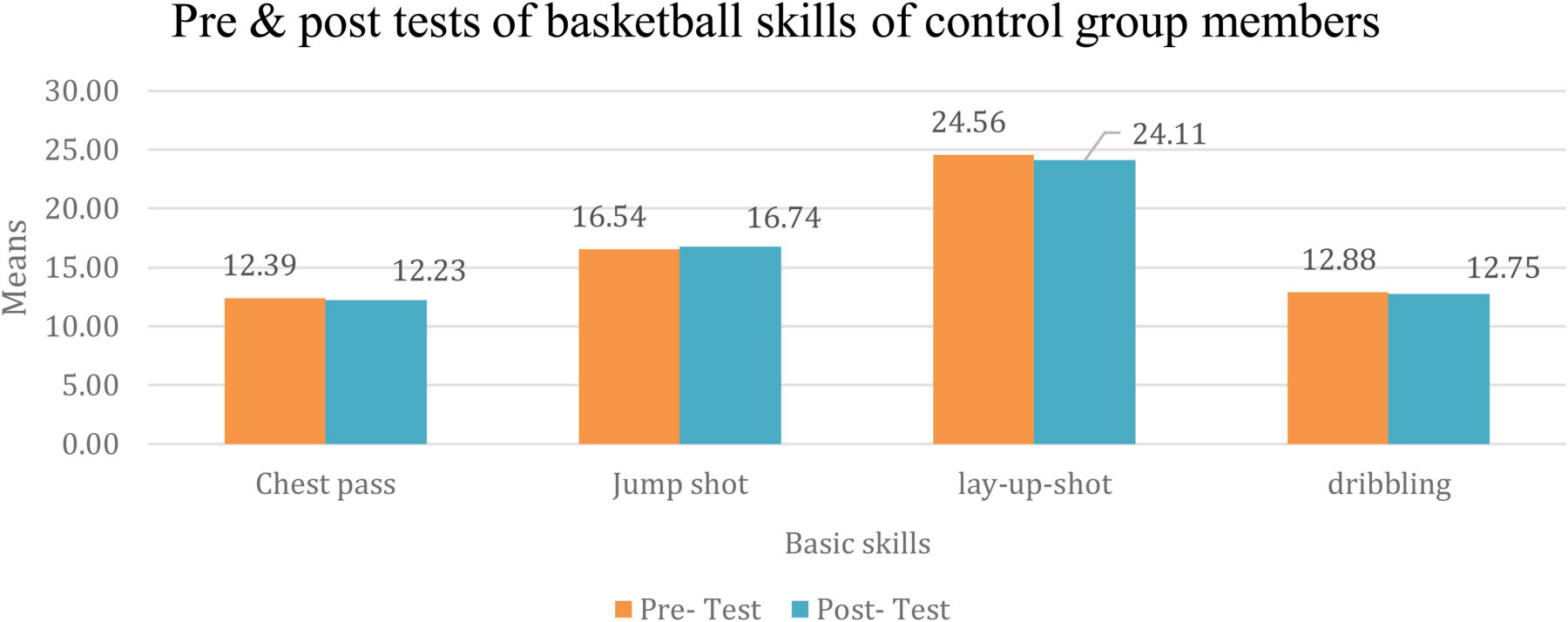
Average pre- and post-measurements for learning basic basketball skills in the control group. Source(s): Authors’ work.

It is evident from Table 6 that there are no statistically significant differences at the significance level (*α* ≥ 0.05) regarding the effect of using the traditional program on learning the basic basketball skills (chest pass, jump shot, lay-up shot, and dribbling) among the members of the control group between the pre- and post-measurements. The percentage changes were as follows: (374.6%, 96.9%, 171.4%, 93.3%) respectively. Figure 1 illustrates the difference between the average pre- and post-measurements for learning basic basketball skills in the control group.

The researchers attribute this result to the fact that traditional education at Birzeit University relies heavily on the teacher providing information to students, who then apply it based on the teacher's model. The teacher prioritizes completing the required basic skills within the semester timeframe, which often leads to students learning skills without engagement or enjoyment. Moreover, students do not typically engage in self-directed learning or dedicate time to practice specific skills Jabali et al. (38). It is important to note that skill training alone cannot sufficiently develop basic basketball skills.

### 4.2 Second hypothesis results

The second hypothesis states that there are statistically significant differences at the significance level (*α* ≤ 0.05) between the pre- and post-measurements concerning the effect of using the flipped classroom strategy to learn the basic skills in the game of basketball (chest pass, jump shot, smooth shot, and dribbling) among members of the first experimental group. To test this hypothesis, a Paired t-test (for correlated samples) was conducted. The significance of the differences between the pre- and post-measurement means for the members of the first experimental group presenting in Table 7 below.

**Table 7:** Paired *t*-test results to assess the impact of the flipped classroom strategy on learning basic basketball skills among members of the first experimental group (*n* = 22).

Basic skills	Unit of measurement	Pre-measurement		Dimensional measurement		t-value	Significance level	Improvement rate%	Cohen's d
		Arithmetic mean	Standard deviation	Arithmetic mean	Standard deviation				
Chest pass	Second	12.86	0.70	9.75	0.82	14.72	*0.000	547.0	0.991
Jump shot	level	16.30	0.75	22.46	0.78	-26.953	*0.000	196.0	1.071
Lay-up-shot	Second	24.49	0.85	18.97	0.52	26.975	*0.000	341.6	0.958
Dribbling	Second	12.95	0.68	7.97	0.61	23.488	*0.000	216.6	0.995

Source(s): Authors’ work.

**Figure 2:**
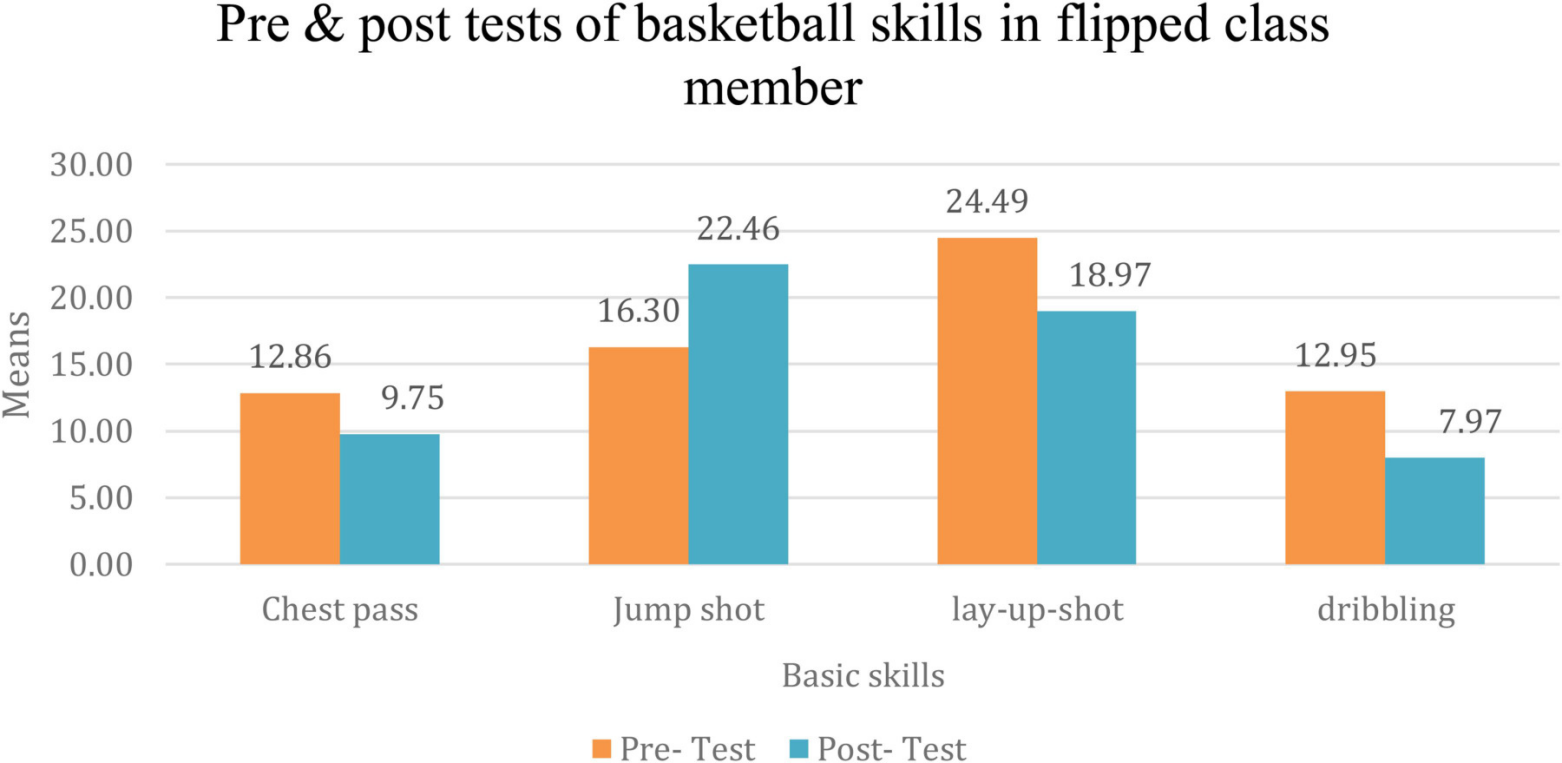
Pre- and post-measurements for learning basic basketball skills among the members of the first experimental group. Source(s): Authors’ work.

It is evident from Table 7 that there are statistically significant differences at the significance level (*α* ≤ 0.05) in the effect of using the flipped classroom strategy on learning basic basketball skills among the members of the first experimental group, between the pre- and post-measurements. The differences are in favor of the post-measurement, with percentage changes of (547.0%, 196.0%, 341.6%, 216.0%) for the chest pass, jump shot, smooth shot, and dribbling skills, respectively. The Figure 2 illustrates the differences in the averages of the pre- and post-measurements for learning basic basketball skills among the members of the first experimental group.

The researchers attribute this result to the fact that, with technological advancements and the challenges the world, and Palestine in particular, has faced including COVID-19, unstable security conditions, and the disruption of face-to-face teaching it became essential to use the internet as a means of overcoming these obstacles. Education through the flipped classroom has provided solutions, allowing students to watch and analyze skills online and then apply them under the guidance of their teacher at the university. As noted by Madhur et al. (39) virtual classrooms, platforms, and other tools enabled learners to easily use computers and smart devices, thereby enhancing the effectiveness of education.

### 4.3 Third hypothesis results

#### 4.3.1 The third hypothesis posits

There are statistically significant differences at the significance level (α ≤ 0.05) in the pre- and post-measurements regarding the effect of using the learning strategy through play on learning the basic skills in basketball (chest pass, jump shot, smooth shot, and dribbling) among members of the second experimental group. To test this hypothesis, a t-test for correlated samples (Paired-t-test) was applied to determine the differences between the means of the pre- and post-measurements for members of the second experimental group. These results are presenting in Table 8.

**Table 8:** Paired *t*-test Results to Assess the impact of the playful learning strategy on learning basic basketball skills among members of the first experimental group (*n* = 22).

Basic skills	Unit of measurement	Pre-measurement		Dimensional measurement		t-value	Significance level	Improvement rate%	Cohen's d
		Arithmetic mean	Standard deviation	Arithmetic mean	Standard deviation				
Chest pass	Second	12.61	0.79	10.95	0.63	7.60	0.000*	304.0	1.028
Jump shot	level	16.36	0.85	18.89	0.56	-13.21	0.000*	116.1	0.898
Lay-up-shot	Second	24.29	0.86	22.46	0.91	7.42	0.000*	145.0	1.155
dribbling	Second	13.01	0.61	11.19	0.57	17.16	0.000*	105.0	0.499

Source(s): Authors’ work.

**Figure 3:**
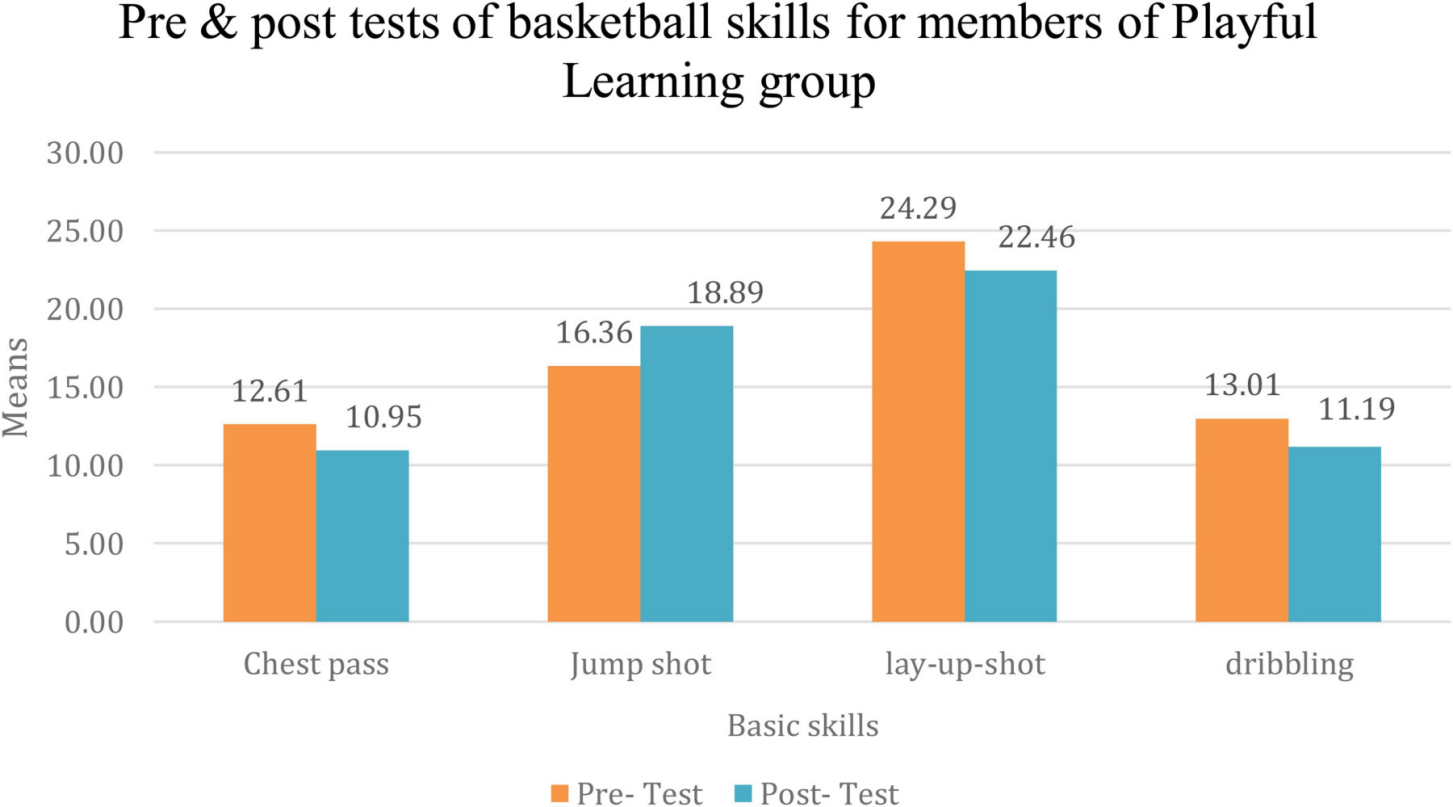
Pre- and post-measurements for learning basic basketball skills among the members of the scend experimental group. Source(s): Authors’ work.

It is clear from Table 8 that there are statistically significant differences at the significance level (0.05 ≥ *α*) on the effect of using the learning strategy from During play On learning the basic skills in the game of basketball (chest pass, jump shot, straight shot, dribbling) among members of the second experimental group between the pre- and post-measurements and in favor of the post-measurement, where the percentage of change was respectively (304.0%, 116.1%, 145.0%, 105.0%) and the Figure 3 shows the difference between the averages of the pre- and post-measurements for learning basic skills in the effect of using the learning strategy through playing, the members of the second experimental group learned the basic skills in basketball (chest pass, jump shot, smooth shot, and dribble).

The researchers attribute this result to the fact that education must be engaging, and learning through play is one of the most effective methods to create an interactive and stimulating learning environment. Parker et al. (36) emphasized that developing learners' abilities depends on their active engagement with methods and strategies, as well as their positive participation to achieve meaningful learning. Yan et al. (40) noted that incorporating play into the learning process of basic basketball skills significantly contributed to the effective acquisition of those skills.

### 4.4 Fourth hypothesis results

#### 4.4.1 The fourth hypothesis states

There are statistically significant differences at the significance level (*α* ≤ 0.05) between the average scores of the students in the control group (taught using the traditional method), the first experimental group (taught using the flipped classroom strategy), and the second experimental group (taught using playful learning) on the basic skills of basketball (chest pass, jump shot, lay-up shot, and dribbling) by dimensional analogy.

To test this hypothesis, arithmetic means and standard deviations were calculated for the performance of the study sample members on the basic skills tests in basketball course 1 (chest pass, jump shot, lay-up shot, and dribbling) across the three groups (control, first experimental, and second experimental) based on the post-test results. These findings are presenting in Table 9.

**Table 9:** Arithmetic means and standard deviations of students’ performance in basic basketball skills tests in the pre- and post-measurements across the control and experimental groups (*n* = 66.)

Basic skills tests	Group	Pre-measurement		Post measurement	
		Arithmetic mean	Standard deviation	Arithmetic mean	Standard deviation
Chest pass	Female officer	12.39	0.78	12.23	0.58
	First Experiment (Flipped Class)	12.86	0.70	9.75	0.82
	The second experiment Playful learning)	12.61	0.79	10.95	0.63
Jump shot	Female officer	16.54	0.77	16.74	0.41
	First Experiment (Flipped Class)	16.30	0.75	22.46	0.78
	The second experiment Playful learning)	16.36	0.85	18.89	0.56
Lay-up-shot	Female officer	24.56	0.77	24.11	0.94
	First Experiment (Flipped Class)	24.49	0.85	18.97	0.52
	The second experiment Playful learning)	24.29	0.86	22.46	0.91
Dribbling	Female officer	12.88	0.48	12.75	0.95
	First Experiment (Flipped Class)	12.95	0.68	7.97	0.61
	The second experiment Playful learning)	13.01	0.61	11.19	0.57

Source(s): Authors’ work

It is evident from Table 9 that there are noticeable differences in the arithmetic means of the scores of the three groups (control, first experimental, and second experimental) on the pre- and post-assessments of the basic skills tests for basketball course 1. To assess the statistical significance of these differences, an analysis of covariance (ANCOVA) was conducted to compare the performance levels of the study sample members on the basic skills tests for basketball course 1, based on the different learning methods (traditional, flipped classroom strategy, and Playful learning strategy). Additionally, Eta squared (*η*^2^) was calculated to determine the effect size of the learning strategies (traditional, flipped classroom strategy, and Playful learning strategy) on the post-test results. These results are presenting in Table 10.

**Table 10:** ANCOVA Results to assess the impact of different teaching strategies (traditional, flipped classroom, playful learning) on the development of basic basketball skills among study sample members (*n* = 66).

Dependent variable	Source of variance	Sum of squares	Levels of freedom	Mean squares	Calculated (F) value	Level Connotation*	ETA square Eta^2^
Chest pass test	Pre-measurement	0.496	1	0.496	1.070	0.305	0.017
	Group	66.536	2	33.268	71.737	0.001	0.698
	Error	28.752	62	0.464			
	Adjusted total	97.149	65				
Jump shot test	Pre-measurement	0.400	1	0.400	1.111	0.296	0.018
	Group	364.291	2	182.146	505.449	0.001	0.942
	Error	22.343	62	0.360			
	Adjusted total	389.292	65				
Lay-up-shot test	Pre-measurement	0.005	1	0.005	0.007	0.932	0.000
	Group	302.883	2	151.441	227.024	0.001	0.880
	Error	41.358	62	0.667			
	Adjusted total	344.253	65				
Dribbling test	Pre-measurement	1.554	1	1.554	3.003	0.088	0.046
	Group	262.993	2	131.497	253.999	0.001	0.891
	Error	32.098	62	0.518			
	Adjusted total	295.521	65				

Source(s): Authors’ work.

The results of Table 10 illustrate that there are statistically significant differences at the significance level (*α* ≤ 0.05) between the arithmetic means of the scores of the three groups (control, first experimental, and second experimental) in the post-measurement of basketball course 1, based on the different learning methods (traditional, flipped classroom strategy, and Playful learning strategy). The F-values for each test were as follows: Chest pass test (*F* = 71.737), Jump shot test (*F* = 505.449), Lay-up shot test (*F* = 227.024), and Dribbling test (*F* = 253.999). These values are statistically significant at (*α* < 0.001), indicating significant differences in the average scores of the three groups on the basic skills tests for basketball course 1 in the post-measurement. These differences can be attributed to the use of flipped classroom strategies and the Playful learning approach during the study. To determine the direction of the differences in average scores among the three groups. These results are presenting in Table 11.

**Table 11:** Adjusted arithmetic means and standard errors for post-test scores on basic basketball skills according to teaching strategies (traditional, flipped classroom, and playful learning).

Basic skills	Group	Adjusted arithmetic averages	Standard error
Chest pass	Female officer	12.259	0.147
	Flipped classroom learning	9.721	0.148
	Playful learning	10.951	0.145
Jump shot	The female officer	16.727	0.129
	Flipped classroom learning	22.465	0.128
	Playful learning	18.897	0.128
Lay-up-shot	Female officer	24.114	0.175
	Flipped classroom learning	18.975	0.174
	Playful learning	22.461	0.175
dribbling	Female officer	12.770	0.154
	Flipped classroom learning	7.966	0.153
	Playful learning	11.168	0.154

Source(s): Authors’ work.

To determine the direction of the differences between the adjusted means, the Bonferroni test was used for multiple dimensional comparisons, and Table 12 presents this.

**Table 12:** Results of using the test Bonferroni (Bonferroni) for the adjusted averages of the scores of the three groups in the post-measurement (*n* = 66).

Basic skills	Groups	Arithmetic average	The three groups		
			Control group	Inverted row	Learning through play
Chest pass	Control group	12.23		*2.539	*1.308
	Flipped classroom strategy	9.75	*2.539-		*1.231-
	Learning strategy by playing	10.95	*1.308-	*1.231	
Jump shot	Control group	16.74		*5.739-	*2.171-
	Flipped classroom strategy	22.46	*5.739		*3.568
	Learning strategy by playing	18.89	*2.171	*3.568-	
Lay-up-shot	Control group	24.11		*5.140	*1.654
	Flipped classroom strategy	18.97	*5.140-		*3.486-
	Learning strategy by playing	22.46	*1.654-	*3.486	
Dribbling	Control group	12.75		*4.805	*1.602
	Flipped classroom strategy	7.97	*4.805-		*3.203-
	Learning strategy by playing	11.19	*1.602-	*3.203	

Source(s): Authors’ work.

The results in Table 12 illustrate statistically significant differences at the *α* ≤ 0.05 level in the post-test between the control group and both the first experimental group (using the flipped classroom strategy) and the second experimental group (using the Playful Learning strategy). These differences favored the flipped classroom strategy and the Playful Learning strategy across all basic skills tests for Basketball Course 1 among physical education students at Birzeit University. Additionally, statistically significant differences were observed at the *α* ≤ 0.05 level between the first and second experimental groups, with the flipped classroom strategy again demonstrating a clear advantage in all basic skills tests for Basketball Course 1. The researchers attribute these results to the flipped classroom strategy, which, alongside the engaging use of technology, stands out in an era where students are accustomed to such tools, especially given the challenges of security concerns and limited in-class time. This strategy enables students to apply what they learn during face-to-face sessions, thereby enhancing their enjoyment and providing ample opportunities for repetition. As Yao et al. (3) noted, the integration of technology in education improves learners’ skills, fosters a competitive environment, saves time and effort, and ensures sufficient feedback (41, 42).

## 5 Conclusion and policy implications

### 5.1 Theoretical and practical implications

The use of modern educational strategies, such as the flipped classroom and gamified learning, significantly enhances the learning of fundamental motor skills, underscoring the need to move away from traditional teaching methods, particularly in practical courses, to improve student experiences. The study highlights the importance of integrating technology and interactive activities into sports education to elevate students' performance and address their individual learning needs.

Researchers recommend adopting the flipped classroom strategy in practical courses, given its positive impact on improving motor skills and allowing more time for hands-on training. Expanding its use across other sports and practical courses is encouraged to enhance educational effectiveness further. Furthermore, lecturers should integrate gamified learning into educational curricula, especially at the foundational stages, as it fosters motivation and engages students in their learning process.

Finally, future research should explore the combination of multiple educational strategies to develop integrated curricula that better meet students' needs and improve the quality of education. The researchers monitored the participants on a weekly basis to collect experimental data for analysis. The results indicate several important implications: The flipped classroom strategy is the most effective approach, followed by project-based learning. Therefore, the flipped classroom strategy is the recommended approach for integrating physical education, specifically basketball, into Palestinian universities.

### 5.2 Conclusion

This study emphasizes the significant impact of modern educational strategies on the development of the educational process, highlighting their role in enhancing the learning experience and improving students' skill performance. The conclusions and recommendations offer valuable insights into the positive influence of strategies like the flipped classroom and Playful learning, while also addressing the challenges faced and suggesting future research directions to further advance the field of sports education.

The study's findings reveal that modern teaching strategies, such as the flipped classroom and Playful learning, are more effective in improving students' basic basketball skills than traditional methods. The flipped classroom strategy, in particular, demonstrated superior results compared to both the Playful learning and traditional approaches, providing students with the opportunity to leverage technology to enhance classroom interaction and optimize time usage. The Playful learning strategy, on the other hand, effectively boosted student motivation and interest, which in turn positively influenced their performance in sports skills.

### 5.3 Limitations and directions for future research

The study's geographical limitation restricts the generalizability of its results, necessitating further studies across broader contexts. Additionally, environmental challenges, such as security interruptions and the short duration of the experiment, hindered the ability to assess the long-term impact of the strategies. Future research should expand to different educational stages or other sports and explore the impact of innovative strategies, such as virtual reality and artificial intelligence, on sports education. Additionally, studies could investigate the effects of combining various educational strategies on enhancing both motor and cognitive performance.

During the study, the researchers adhered to the following parameters: Human Scope: The study involved physical education students enrolled in the second semester of the 2023–2024 academic year. Time Frame: The study was conducted between March 15 and June 15, 2024. Spatial Scope: The study took place in the gymnasium of the Department of Physical Education at Birzeit University.

## Data Availability

The raw data supporting the conclusions of this article will be made available by the authors, without undue reservation.
