# Supplementary material for: Impact of flipped classroom strategy and learning on the acquisition of basic skills among students in the basketball 1 course at Birzeit University
Source: Front Sports Act Living. 2025 Nov 24;7:1660007. doi: 10.3389/fspor.2025.1660007 (PMC12682784; doi:10.3389/fspor.2025.1660007)
Supplement: Supplementary file 1 [file Supplementaryfile1.docx]

**Appendix (1)**

**Skill performance tests registration form**

Student name: ............................................

height: .................................................

Mass: ................................................

| Number | Skill name | Test name | Try | Pre-test | post-test |
| --- | --- | --- | --- | --- | --- |
| 1- | Chest pass (10 passes) | Measure scroll speed | first (second) |  |  |
|  |  |  | second (second) |  |  |
| 2- | Jump shot at the basket (15 shots) | Measuring aiming accuracy | First (level) |  |  |
|  |  |  | second (level) |  |  |
| 3- | Lay-up-shot | Measuring the accuracy of a lay-up-shot | first (second) |  |  |
|  |  |  | second (second) |  |  |
| 4- | Dribbling | Measuring the speed of dribbling in a zigzag manner | first (second) |  |  |
|  |  |  | second (second) |  |  |

**Appendix (2)**

**2.1 Accuracy of the chest pass:**

- Purpose of the test: to measure scrolling speed.

- Tools used: a legal basketball, a smooth wall perpendicular to the ground, a stop watch.

Performance Specifications:

A- A smooth wall perpendicular to the ground. A line parallel to the wall and at a distance of (270 cm) is drawn on the ground. The player stands with the ball behind the starting line, then passes the ball towards the wall (10) chest passes.

B- The time is calculated from the moment the start signal is given until the tenth ball touches the wall.

Calculating grades:

- The number of seconds it takes a player to perform (10) chest passes.

-The player is not allowed to move more than one step in any direction. If the player moves more than one step, the test will be repeated.

Each laboratory is given two attempts to perform the test, and the best one is counted. Figure 1 shows this.


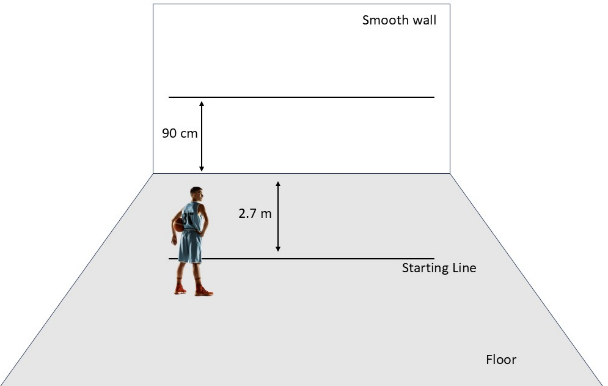


**Figure 1.** Accuracy of the chest pass

Source(s): Authors’ work

**2.2 jump shot:**

- Purpose of the test: to measure the accuracy of jump shot.

- Tools used: basketball court, basketball, basketball goal

Necessary procedures: Draw three dots in the form of a small circle with a diameter of 15 cm as signs indicating the three areas through which the test is taken.

A- The first mark is to the left of the end of the free throw line and is 30 cm away from it.

B- The second mark is in the middle of the free throw line and 90 cm away from the far scoring line (three points).

C- The third mark to the right of the end of the free throw line and at a distance of (30 cm).

- Performance description:

- Each player is given (15) shots to perform, distributed among the three marks in the middle of the free throw line, so that in each mark he performs (5) jump shots.

Each laboratory is awarded two points for each ball that enters the basket without touching the ring, and one point for each ball that touches the ring and does not enter the basket. The laboratory’s grades are the sum of the points obtained from shooting from the three marks and the maximum score for the test (30 marks), and Figure 2 shows that.


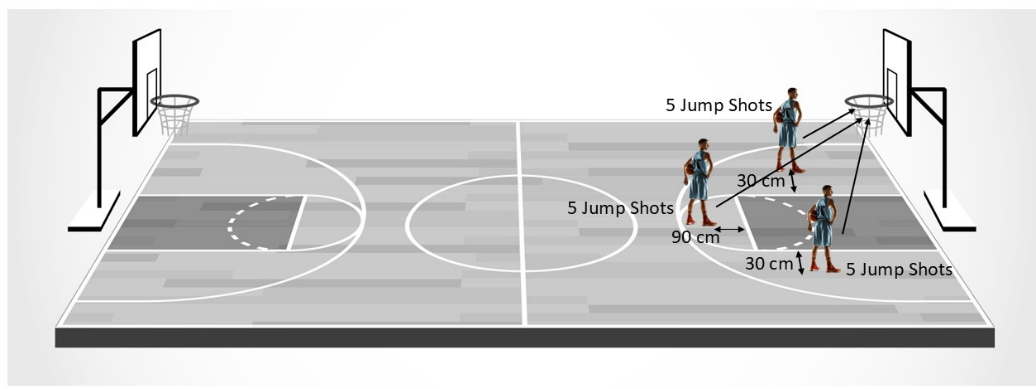


**Figure 2.** Jump Shot

Source(s): Authors’ work

**2.3 lay-up-shot test**

The purpose of the test is to measure the accuracy of a lay-up-shot

- Tools used: basketball, basketball court, signs, stopwatch, (5) signs

- Description of the performance: The player stands on the starting line, which is (19.5 m) away from the target, and when he hears the start signal, he dribbles between the signs that are (1.50 m) apart from each other. When he passes the last sign, he takes a safe shot. If the shot is successful, the tester takes a shot. The ball and quickly return to the starting line. If the safe shot is not successful, the player continues to try until he succeeds in performing the safe shot. Then he takes his ball and quickly returns to the finish line, and then the clock is stopped. Timing.

- Recording: The player is given two attempts to perform and the time of the best attempt is recorded for him, and Figure 3 shows this.


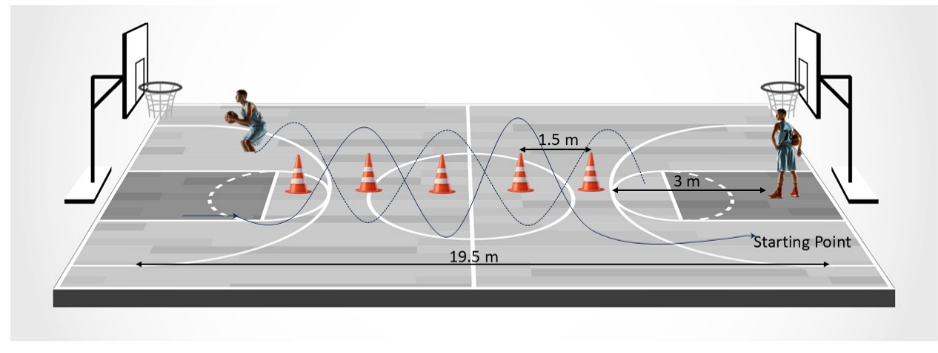


**Figure 3.** Lay-up-shot test

Source(s): Authors’ work

**2.4 Dribbling:**

- Purpose of the test: to measure the speed of dribbling in a zigzag manner.

- Tools used: basketball, stop watch, basketball court, (6) signs.

- Procedures: Draw a line for the start and end, and the first sign is placed at a distance of (1.50 m) from the starting point, and the distance between the signs is (2.40 m).

- Description of the performance: The player stands with the ball in his hand behind the starting line, and when the signal is given to start, the player dribbles and runs as quickly as possible between the barriers forward, and when he reaches the finish line, he turns around and returns to the starting line again.

- Recording: The time spent by the player during the performance is recorded, and Figure 4 shows this.


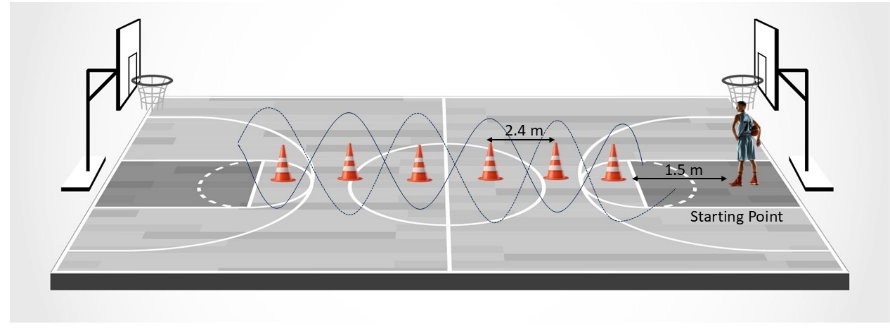


**Figure 4.** Dribbling

Source(s): Authors’ work

**Appendix (3)**

**Appendix (3.1) The regular educational program (control).**

**The researchers conduct a joint warm-up between the three groups.**

By explaining the technical aspects of the skill, doing a model in front of the group, identifying technical errors, applying the skill with simple exercises and then complex exercises, dividing the students into groups, then the teacher distributes the group into four equal groups and plays a competition game between the groups and emphasizes the learned skill.

**Appendix (3.2) The educational program using the flipped classroom strategy (first experimental)**

The researchers will implement the educational program using the flipped classroom, so that a website will be created on the social networking site (Facebook) or through Retaj or (ITC) for the first experimental group after all members of the experimental group have been involved in this work, and an educational video on basic skills will be prepared tomorrow. The basket in which they will be tested (chest pass, jump shot, lay-up, dribbling) (and sending it to the group and informing them of the date of publication, sharing their opinions, writing notes and questions, and communicating. Among themselves before attending the lesson, and during the lecture, the student applies what he or she saw in the video with the participation of both the teacher and fellow student. Finally, the teacher and student discuss the questions that were recorded.

**Appendix (3.3) The educational program using the learning strategy by playing. (Second experimental)**

The teacher gives lessons in the gamified learning style with the second experimental group, where the researchers designed the educational program for the gamified learning strategy by designing small games that the students in equal groups play and implement, aiming to develop basic basketball skills.

Note that the three groups, the control, the first experimental, and the second experimental, share the educational curriculum in teaching the same basic basketball skills on the same court.

**Appendix (4)**

**Tutorial using the flipped classroom strategy to teach basketball skills (for 3 months)**

| **month** | **week** | **Target skill** | **Educational content** | **Practical content** | **Comments** |
| --- | --- | --- | --- | --- | --- |
| the first | 1+2 | Lay-up-shot | - Watch educational videos about lay-up-shot | - Practice correct posture and timing. | - Short tests on basic grammar. |
|  |  |  | - Read texts that explain the dynamics of movement. | - Individual guidance to improve performance. | - Analyzing students’ performance using video. |
|  | 3+4 |  |  | - Divide students into small groups to practice lay-up-shot. | - Small competitions to motivate students to progress. |
| The second month | 1+2 | Jump shot | - Videos explaining the difference between lay-up-shot and jump shot. | - A practical application that focuses on balance while jumping. | - Video evaluation of each student’s performance and analysis of technical points. |
|  | 3+4 | Dribbling | - Watch videos learning to control the ball while dribbling. | - Drills using cones to improve ball control. | - Notes from the trainer for each student based on his performance during the interview. |
| The third month | 1+2 | Chest pass | - A theoretical explanation using videos about the technical steps of passing. | - Training in pairs and at different distances to develop accuracy and strength. | - A practical test to evaluate the accuracy and strength of the chest pass. |
|  | 3+4 | Integration of skills | - A theoretical review of the four skills and linking them to their application in matches. | - A practical application that integrates skills: lay-up-shot, jump shot, dribbling, and passing. | - Holding small matches to evaluate the use of skills in an integrated manner. |

Source(s): Authors’ work

**Appendix (5)**

**The panel of experts who evaluated the suitability of the tests to the study's objectives**

| name | Qualifications | Affiliations | Expertise/experience |
| --- | --- | --- | --- |
| Islam Abbas | Associate Professor - PhD in Physical Education from the University of Jordan | Arab American University | Basketball, sports training, motor skills development |
| Hashem Al-Kilani | Professor - Ph.D., University of Illinois | Birzeit University - Master's Program in Physical Education and Sports Science | Kinesiology - Basketball Sports Training |
| Alaa’ Kamal | Associate Professor - PhD in Physical Education | Palestine Technical University-Kadoorie | Sports training and training programs |
| Awad Bdair | Assistant Professor - PhD in Physical Education | Palestine Technical University-Kadoorie | Sports training, basketball |
| Mohannad Omar | Assistant Professor - PhD in Physical Education | Palestine Technical University-Kadoorie | Sports training, sports supervision |
| Ziad Ermeli | Assistant Professor - PhD in Physical Education | University of Jordan | Football sports training |

Source(s): Authors’ work
